# A descriptive analysis of antimicrobial resistance patterns of WHO priority pathogens isolated in children from a tertiary care hospital in India

**DOI:** 10.1038/s41598-021-84293-8

**Published:** 2021-03-04

**Authors:** Vijayalaxmi V. Mogasale, Prakash Saldanha, Vidya Pai, P. D. Rekha, Vittal Mogasale

**Affiliations:** 1 Department of Paediatrics, Yenepoya Medical College, Mangalore, India; 2 Department of Microbiology, Yenepoya Medical College, Mangalore, India; 3 Yenepoya Research Centre, Yenepoya (Deemed to be) University, Mangalore, India; 4 Policy and Economic Research Department, International Vaccine Institute, Seoul, South Korea

**Keywords:** Antimicrobials, Clinical microbiology, Policy and public health in microbiology, Microbiology, Health care, Health policy, Health services, Paediatrics, Public health

## Abstract

The World Health Organization (WHO) has articulated a priority pathogens list (PPL) to provide strategic direction to research and develop new antimicrobials. Antimicrobial resistance (AMR) patterns of WHO PPL in a tertiary health care facility in Southern India were explored to understand the local priority pathogens. Culture reports of laboratory specimens collected between 1st January 2014 and 31st October 2019 from paediatric patients were extracted. The antimicrobial susceptibility patterns for selected antimicrobials on the WHO PPL were analysed and reported. Of 12,256 culture specimens screened, 2335 (19%) showed culture positivity, of which 1556 (66.6%) were organisms from the WHO-PPL. *E. coli* was the most common organism isolated (37%), followed by *Staphylococcus aureus* (16%). Total of 72% of *E. coli* were extended-spectrum beta-lactamases (ESBL) producers, 55% of *Enterobacteriaceae* were resistant to 3rd generation cephalosporins due to ESBL, and 53% of *Staph. aureus* were Methicillin-resistant. The analysis showed AMR trends and prevalence patterns in the study setting and the WHO-PPL document are not fully comparable. This kind of local priority difference needs to be recognised in local policies and practices.

## Introduction

Antimicrobial resistance (AMR) has been recognised as a major threat to global health^1^. According to the World Health Organization (WHO), mutations in microorganisms resulting in AMR, which consequently render medicines ineffective and infections persist in the body, increasing the risk of spread to others^1^. There are many reasons behind the development of AMR, ranging from microbial causes to human aspects such as overuse and over-prescription of antimicrobials, agricultural and commercial application of antimicrobials in the animal sector, and human behavioural factors^2^. Our ability to treat common pathogens becomes challenging because of AMR, resulting in increased duration of illness, costs, number of complications, and deaths. By 2050, an estimated 10 million deaths are projected to occur due to AMR^3^, while another study projected AMR to cost the global economy US$100 trillion, in the same period^4^.

**Figure 1:**
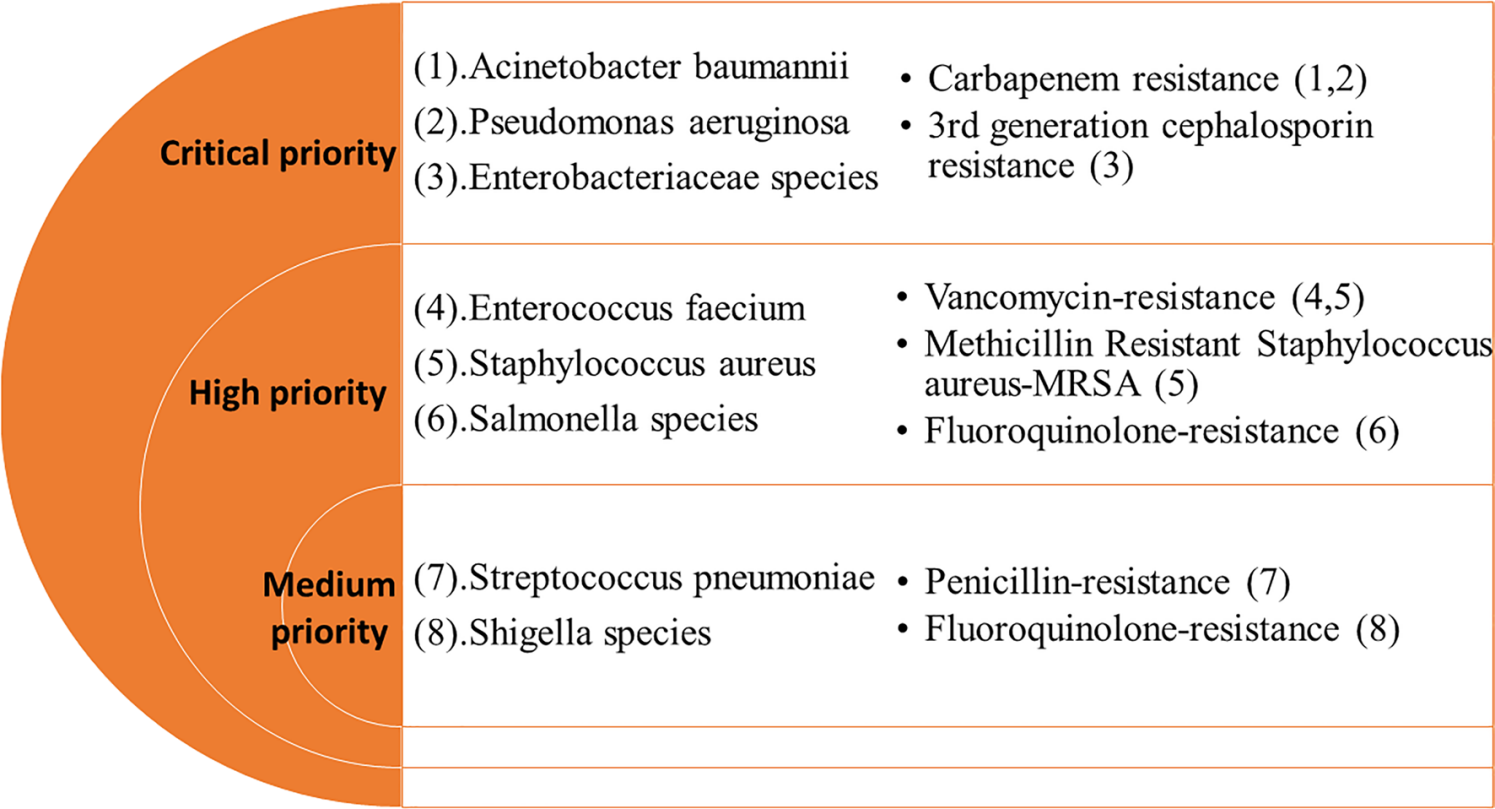
The list of organisms and antimicrobial resistance patterns included in the analysis based on the World Health Organization priority pathogens list (WHO PPL).

In 2015, the 68th World Health Assembly endorsed the Global Action Plan on AMR to tackle this global challenge^5^. This action plan has five strategic actions, focusing on (1) improving awareness and understanding of AMR; (2) strengthening AMR surveillance; (3) reducing the incidence of infections; (4) optimizing antimicrobial use; and (5) developing the economic case for AMR control. To support the Global Action Plan, WHO has developed a priority pathogens list (PPL), through a consultative process^6^. The prioritization process involved multi-criteria decision analysis (MCDA) which used information from multiple sources, including disease mortality, transmissibility, treatability, health care burden, preventability in health care settings, and preventability in community settings, etc. Twelve families of drug-resistant bacteria, posing the greatest threat to human health, were categorized as critical, high, and medium priority organisms, in terms of their resistance to selected antimicrobials (Fig. 1). Although this categorization was intended to prioritize and stimulate research and develop new antimicrobials for specific drug resistance, it also makes a call for the prevention of infection and the rational use of antibiotics in both humans and animals^6^.

Indian population is known to be the highest consumer of antibiotics in the world^7^. The AMR situation in India has raised grave public health concerns^8^ and an action plan for its control is considered crucial^9,10^. Given its importance for human health, the Government of India has developed a National Action Plan on Antimicrobial Resistance (NAP-AMR) 2017–2021^11^. Strengthening the knowledge and evidence base through surveillance of AMR is one of the five key strategies of this action plan. The Indian Council of Medical Research (ICMR) has established an Antimicrobial Resistance Surveillance & Research Network (AMRSN) across selected hospitals in India, focusing on drug resistance among six pathogens^12^. However, not many hospitals outside this network in India track AMR patterns among these pathogens. Generating AMR related evidence from a larger number of hospitals is critical for informed decision making on AMR related policies and practices at local settings.

This study explores the AMR susceptibility patterns for WHO priority pathogens identified in clinical isolates collected in the Paediatrics Department of a tertiary care hospital in Southern India. The results of the culture tests are mainly availed for treatment purposes but are not systematically analysed on a routine basis. The analysis of culture results could provide further evidence and guidance for the development of antimicrobial resistance control policy at the hospital and elsewhere. This analysis aims to compare AMR patterns in WHO priority pathogens identified in a tertiary health care facility to understand the local priorities that can be applied to local policies and practices.

## Results

A total of 12,256 culture specimens collected at paediatrics outpatient department and paediatrics inpatient wards were screened for bacteriological results, of which 2335 (19%) showed culture positivity. Of these, 1556 were from the set of WHO PPL organisms. The largest number of bacterial isolation was seen in urine specimens (755/1556) followed by blood (241/1556) (Table 1). *E. coli* was the most common organism isolated (576), followed by *Staphylococcus aureus* (252).

**Table 1:** World Health Organisation (WHO) Pathogen Priority List (PPL) organisms isolated in the study site by specimen type from January 2014 to October 2019.

Specimen	Blood	Urine	Stool	Pus	Sputum	CSF and sterile fluids	Aspirate	Swab	Tissue	Central lines and stents	Catheter tip	ET tip	Other instruments	Total
**Critical priority organisms**														
*Acinetobacter baumannii*	6	17	0	1	3	1	2	4	0	6	11	10	4	65
*Pseudomonas aeruginosa*	5	23	0	7	6	1	3	6	1	31	16	16	2	117
*Enterobacteriaceae*														
* E. coli*	13	447	29	28	5	4	2	8	8	4	15	7	6	576
* Klebsiella* spp.	35	124	4	7	11	2	2	7	1	10	13	13	0	229
* Enterobacter* spp.	31	53	4	4	3	2	3	5	2	7	38	6	4	162
* Serratia* spp.	0	17	0	1	1	0	1	0	0	1	5	3	1	30
* Proteus* spp.	0	21	0	3	0	1	0	1	0	0	0	0	1	27
* Providencia* spp.	0	5	0	0	0	0	0	0	0	0	0	0	0	5
* Citrobacter* spp.	0	16	0	1	2	0	0	1	0	3	12	6	5	46
* Morganella* spp.	0	8	0	0	0	0	0	0	0	0	0	0	0	8
* Others* spp.	0	2	0	0	0	0	0	0	0	1	2	1	0	6
**High priority organisms**														
*Enterococcus faecium*	5	9	0	0	0	0	0	0	0	0	1	0	0	15
*Staphylococcus aureus*	135	12	0	66	2	0	1	26	0	2	2	4	2	252
*Salmonella species*	8	0	0	0	0	0	0	0	0	0	0	0	0	8
**Medium priority organisms**														
*Streptococcus pneumoniae*	3	0	0	0	1	0	0	2	0	0	0	0	0	6
*Shigella species*	0	1	3	0	0	0	0	0	0	0	0	0	0	4
Total organisms	241	755	40	118	34	11	14	60	12	65	115	66	25	1556

*CSF* cerebrospinal fluid, *ET* endo tracheal.

Among the main WHO PPL organisms identified, 72% of *E. coli* and 63% of *Klebsiella* spp. were resistant to 3rd generation cephalosporins due to extended-spectrum beta-lactamase (ESBL), and 53% of the *Staph. aureus* were Methicillin-resistant (Table 2). Overall, nearly half of *Enterobacteriaceae* were resistant to carbapenem (46%) or 3rd generation cephalosporins due to ESBL (55%). The carbapenem resistance in *Pseudomonas aeruginosa* was found low (5%).

**Table 2:** Selected antimicrobial resistance in World Health Organisation (WHO) Pathogen Priority List (PPL) organisms isolated in the study from January 2014 to October 2019.

Critical priority organisms	Isolated (n)	Tested (n)	Resistant (n)	Resistant (%)	Tested (n)	Resistant (n)	Resistant (%)
		Carbapenem-resistant			3rd generation cephalosporin-resistance due to ESBL		
***Enterobacteriaceae***	1089	606	276	45.54	721	396	54.92
*E. coli*	576^a^	342	140	40.94	410	297	72.44
*Klebsiella* spp.	229^a^	107	58	54.21	133	84	63.16
*Enterobacter* spp.	162^a^	103	59	57.28	111	8	7.21
*Serratia* spp.	30^a^	10	9	90.00	10	2	20.00
*Proteus* spp.	27^a^	15	1	6.67	20	0	0.00
*Providencia* spp.	5^a^	4	1	25.00	5	0	0.00
*Citrobacter* spp.	46^a^	13	6	46.15	19	4	21.05
*Morganella* spp.	8^a^	6	0	0.00	7	1	14.29
*Others* spp.	6^a^	6	2	33.33	6	0	0.00
***Acinetobacter baumannii***	65	52	6	11.54			
***Pseudomonas aeruginosa***	117	59	3	5.08			

High priority organisms		Tested	Resistant	Percentage (%)	Tested	Resistant	Percentage
		Vancomycin-resistant			Methicillin-resistant *Staphylococcus aureus* (MRSA)		
*Staphylococcus aureus*	252	174	3	1.72	188	99	52.66
*Enterococcus faecium*	15	15	2	13.33			
		**Fluoroquinolone-resistant**					
*Salmonella species*	8	4	3	75.00			

Medium priority organisms		Tested	Resistant	Percentage (%)			
		Fluoroquinolone-resistant					
*Shigella species*	4	4	3	75.00			
		**Penicillin-resistant**					
Streptococcus pneumoniae	6	6	2	33.33			
Total organisms	1556						

*ESBL* extended-spectrum β-lactamases.

^a^These numbers are already counted in the subtotal.

**Figure 2:**
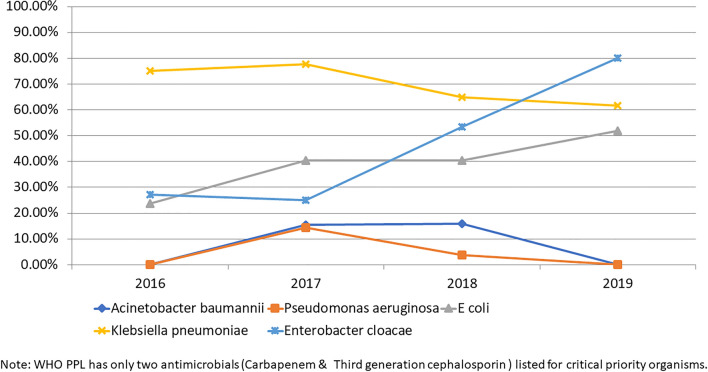
Carbapenem-resistance trends among selected WHO Critical Priority Pathogens.

Time trend analysis of selected WHO ‘Critical priority’ pathogens over the past 4 years showed a high proportion of resistance for carbapenem in *E coli*, *Klebsiella pneumoniae,* and *Enterobacter cloacae* (Fig. 2).

**Figure 3:**
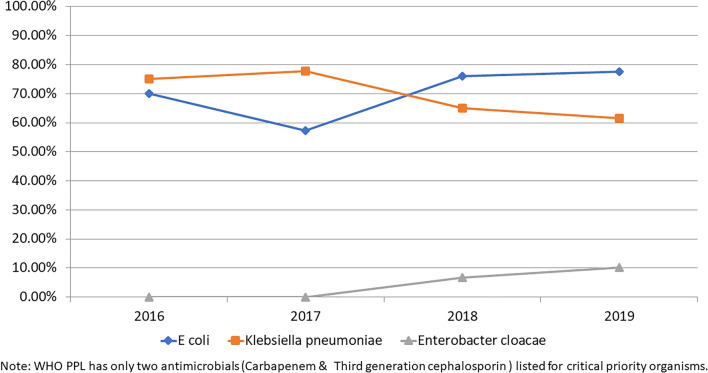
Third generation cephalosporin-resistant trends (due to extended-spectrum beta-lactamases (ESBL)) among selected WHO Critical Priority Pathogens.

Similarly, *E. coli* and *Klebsiella pneumoniae* continued to show a high proportion of ESBL (Fig. 3).

**Figure 4:**
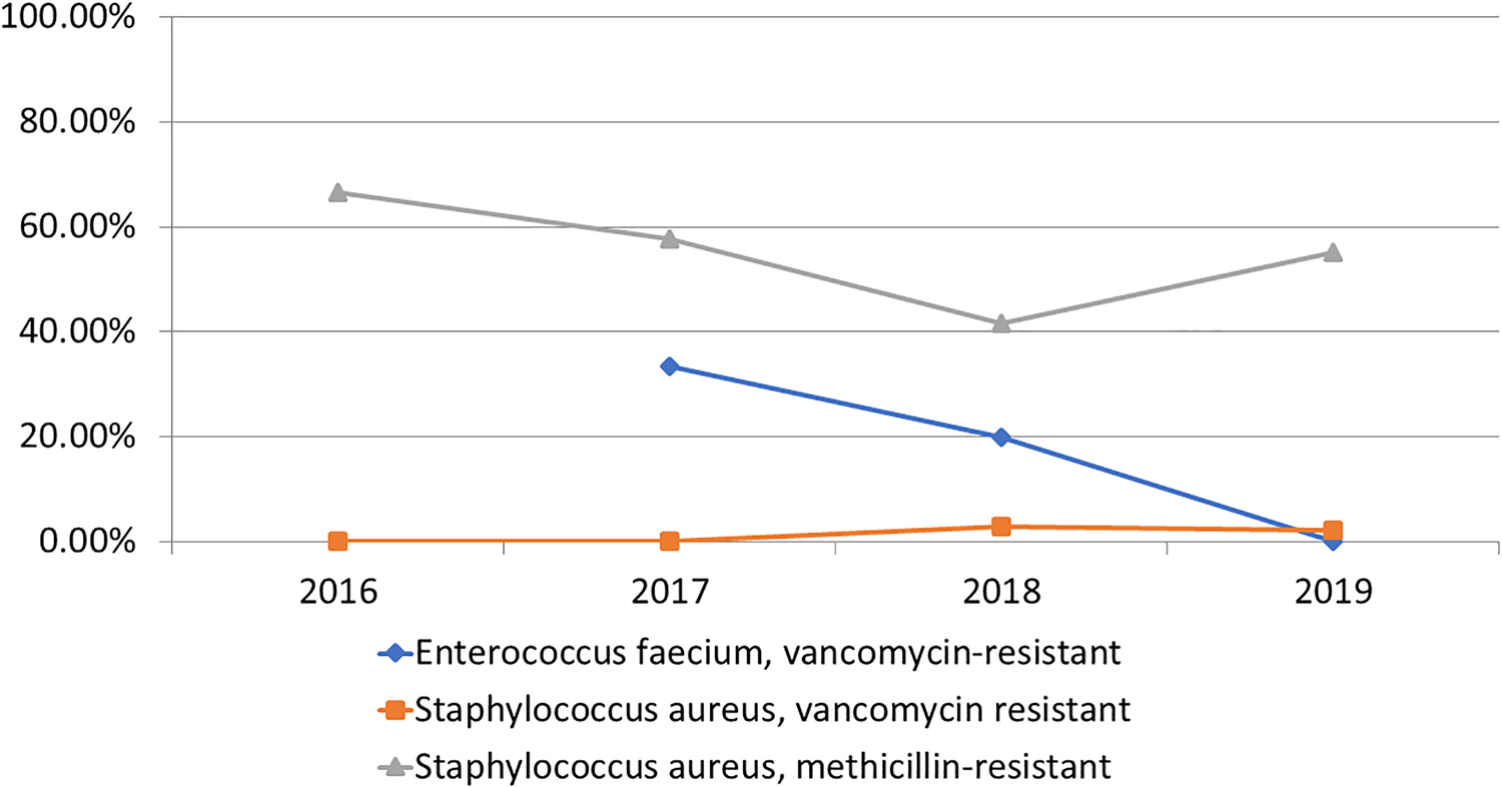
Resistance trends among selected WHO High Priority Pathogens.

Among the WHO ‘High priority’ pathogens, *Staph. aureus* continued to show a high proportion of methicillin-resistance (Fig. 4).

Three of the WHO ‘High priority’ pathogens namely, *Helicobacter pylori* (clarithromycin- resistant), *Campylobacter* spp. (fluoroquinolone-resistant), and *Neisseria gonorrhoeae* (3rd generation cephalosporin-resistant and fluoroquinolone-resistant), were not detected in our study specimens and antimicrobial sensitivity information was not available. Similarly, one of the WHO ‘Medium priority’ pathogen, *Haemophilus influenzae* (ampicillin-resistant), was not observed in this study, and antimicrobial sensitivity information was not available.

## Discussion

The 2017 guidance document of WHO indicated the highest carbapenem resistance worldwide in *Acinetobacter baumannii* (91%) and *Pseudomonas aeruginosa* (82%), which is one of the reasons for classifying them as Critical Priority^6^ pathogens. The same study reported > 50% carbapenem resistance in *Acinetobacter baumannii*, and 31% to 50% carbapenem resistance in *Pseudomonas aeruginosa* in the Indian sub-continent in the general population. Early results from the surveillance data from up to 22 ICMR-AMRSN sites in India showed around 80% carbapenem resistance in *Acinetobacter baumannii* and around 30% in *Pseudomonas aeruginosa*^12^. However, another study in children from Mumbai, India, identified only 15% of *Pseudomonas aeruginosa* are resistant to carbapenem^13^ which is comparable to current study. The carbapenem-resistance in *Acinetobacter baumannii* and *Pseudomonas aeruginosa* are 12% and 5% respectively in the current study in paediatric population.

The WHO report^6^ identified high carbapenem resistance in *E. coli* (55%), *Klebsiella* (70%), and *Enterobacter* spp. (59%) in the general population, which is not far apart to the findings in this study in paediatric population. The ICMR-AMRSN data also showed similar carbapenem resistance prevalence in *Klebsiella pneumoniae* (40–50%) but a significantly lower level in *E. coli* (15–25%) in the general population^12^. The ESBL trends in *E. coli* and *Klebsiella* spp. (70–80%) as well as methicillin-resistance in *Staph. aureus* (53%) were high in this study and comparable to the WHO report. However, due to the low sample size of *Salmonella* spp., *Shigella* spp. and *Streptococcus pneumoniae*, it may not be appropriate to compare the results from this study to others.

Several studies on AMR have been published in India in recent years^13–15^. A retrospective 5 year follow-up study in a tertiary care hospital in North India showed increasing trends of AMR in urinary tract infection-causing isolates^14^. Increasing trends of AMR was observed among gram negative isolates from samples collected across seven hospitals in India over 4 years, but the reported carbapenem resistance prevalence in *Klebsiella* spp. (39%) and *E. coli* (12%) were lower than current study^15^. Among *Enterobacteriaceae* isolated from a paediatric tertiary care hospital in Mumbai, 24% were extended spectrum beta-lactamase (ESBL) producers and 27% were carbapenem-resistant isolates showing a lower resistance level than the current study^13^.

As our study is based on a retrospective dataset, it has several limitations. Although the health facility maintains a good quality of clinical and laboratory services along with proper documentation, one cannot ensure that the quality checks in retrospective data are fully compatible with the highest quality standards of a well-conducted prospective study. Although the sample collection, microbiological analysis, and report updates use standard procedures, it has likely been conducted by different people over the 5 year period, which may have had inter-personnel variations on the quality of laboratory procedures. It is possible that at any given point of the study period new laboratory staff may have joined, may have taken time while undergoing training to implement standardized procedures. Also, laboratory reports are manually entered into computerized system which is subject to human error and specific terms used during data entry are subject to human variations. There were no standard inclusion criteria for sample collection as it was generally left to the discretion of the treating physician.

In conclusion, among the WHO PPL pathogens, *E. coli, Klebsiella species*, *Enterobacteriaceae, and Staph. aureus* (methicillin-resistant) have high AMR in the study site. On the other hand, AMR patterns for *Acinetobacter baumanni*, *Pseudomonas aeruginosa,* and *Staph. aureus* (vancomycin resistant) is lower than the WHO global estimates. These findings can guide local priorities, policy, and practices. We recommend large health facilities to monitor and review emerging AMR patterns and trends periodically to prioritise, plan, and implement health facility level policies and guidelines for the optimal use of antimicrobials.

## Methods

The study was conducted at the Yenepoya Medical College Hospital in Mangalore, South India. Typically, the Paediatric Department collected around 2000 clinical specimens every year for culture tests from both outpatient and hospitalized cases. The sources and types of specimens collected for culture included blood, urine, stool, pus, cerebrospinal fluid (CSF), sputum, and any other bodily fluids or other clinical specimens such as catheter, umbilical, and central line tips. As this is a retrospective study, the sample selection for specimen collection was left to the discretion of the treating physician as sampling criteria was not predefined. The specimens were referred to the laboratory in the Department of Microbiology for antimicrobial culture tests and antibiogram. The Kirby-Bauer disk diffusion method and/or by BD phoenix automated system were used for performing antimicrobial susceptibility patterns and reported according to standard (Clinical Laboratory Standards Institute—CLSI) guidelines^16–20^. The confirmation of ESBL was done as per the same CLSI guidelines. Once tests were performed, results were entered into the computer backbone system at the Department of Microbiology which was a specific database for the tertiary hospital included in the study. The antibiogram reports generally covered the following antimicrobials: Carbapenem, Chloramphenicol, Cotrimoxazole, Nitrofurantoin, Piperacillin, Piperacillin-Tazobactam, Tetracyclin, Tigecycline, Aztreonam, Amikacin, Gentamycin, Tobramycin, Ciprofloxacin, Levofloxacin, Norfloxacin, Cefoperazone, Ceftriaxone, Cefotaxime, Ceftazidime, Cefepime, Cefazolin, Cefuroxime, Cefoxitin, Imipenem, Meropenem, Polymyxin B, and Colistin. The sample collection, microbiological analysis, and report entry on the computer were done on a routine basis alongside the provision of healthcare services. These test results for antimicrobial susceptibility patterns were retrospectively accessed through the computer backbone system during this study.

Administrative permission to access laboratory culture records was obtained from Yenepoya Medical College. Retrospective culture reports between 1st January 2014 to 31st October 2019 from various clinical specimens were extracted from the computer backbone system. The culture access numbers for all specimens with positive results were used to track the antibiogram (i.e., which antibiotics were tested, and which were susceptible or resistant) results. The antibiogram for all culture isolates was extracted. Culture access numbers were also used to track the culture source and the date of sample collection to the antibiograms.

Culture reports of all paediatric cases were included in the study, irrespective of the location of sample collection, namely: outpatients, inpatient wards, and neonatal and paediatric intensive care units (ICUs). The laboratory reports indicating contamination were excluded at data entry. The reports containing duplicate or repeat samples, from the same source and subject, were also excluded so that the results for the same pathogens are not duplicated or repeated in the analysis. Pathogens other than those in the WHO PPL were excluded from the analysis. The antimicrobial sensitivity tests other than the ones listed in WHO PPL were also excluded from the analysis. Some of the WHO PPL pathogens were not included in this study as culture specimens were collected from children which do not generally include genitourinary swabs or gastric biopsy specimens suitable for the culture of *Neisseria gonorrhoeae* or *Helicobacter pylori*. Similarly, *Campylobacter* spp. was also not identified in the specimens either because of low incidence or because samples collected may not be best suited for its isolation.

The data entry and analysis were performed on Microsoft Excel. The data from the computer backbone was entered directly on a master Excel sheet followed by the removal of duplicates. De-identified data were organised by specimen types (such as blood, urine, etc.) in chronological order of specimen collection date. A list of WHO PPL bacterial pathogens isolated were prepared by their species (such as *E coli*), specimen type, and resistance patterns. The list classified pathogens into three main groups (Critical Priority, High Priority, and Medium Priority) for selected antimicrobials, based on WHO PPL (Fig. 1). The PPL defines the priority of pathogens based on resistance to specific antimicrobials such as carbapenems, 3rd generation cephalosporins, vancomycin, methicillin, penicillins, or fluoroquinolones. A table presenting the number of organisms isolated in the study site by specimen type was prepared. Another table was prepared to present selected AMR patterns in specific pathogens as defined by WHO PPL. Time trend graphs were prepared for some key pathogens.

### Ethical issues

This study did not involve human subjects directly. An approval from the scientific and ethics committees of Yenepoya Medical College (Name: *Yenepoya Ethics Committee-1*) was obtained for this study. The *Yenepoya Ethics Committee-1* waived the need for participants to provide informed consent. To maintain confidentiality, no identifiable information such as names, addresses, or phone numbers of subjects were collected. The data set, once finalised, was delinked from culture access numbers before analysis, to retain confidentiality.

### Ethics approval and consent to participate

The *Yenepoya Ethics Committee-1* waived the need for participants to provide informed consent as described under the manuscript. This study did not involve human subjects directly, no consent process was involved. All methods were carried out in accordance with relevant guidelines and regulations.

### Consent for publication

All authors have consented for publication.

## Data Availability

All the data included in the manuscript.
